# Prevalence of allergic rhinitis symptoms and associated factors in six-year-old children in a municipality in southern Brazil

**DOI:** 10.1590/1980-549720230024

**Published:** 2023-05-05

**Authors:** Manuela Silva e Silva, Jefferson Traebert, Daniel José da Silva, Eliane Traebert

**Affiliations:** Universidade do Sul de Santa Catarina, Faculdade de Medicina – Palhoça (SC), Brazil.; Universidade do Sul de Santa Catarina, Programa de Pós-Graduação em Ciências da Saúde – Palhoça (SC), Brazil.

**Keywords:** Allergic rhinitis, Allergy, Prevalence, Children, Pediatrics, Rinite alérgica, Alergia, Prevalência, Crianças, Pediatria

## Abstract

**Objective:**

To estimate the prevalence of allergic rhinitis symptoms and associated factors in six-year-old children.

**Methods:**

Cross-sectional epidemiological study involving 956 six-year-old schoolchildren from Palhoça, Santa Catarina, Brazil. Home interviews were conducted with mothers in which socio-demographic and house environmental conditions information were obtained, and the International Study of Athma and Allergies in Childhood (ISAAC) questionnaire for allergic rhinitis symptoms was applied. Bivariate and multivariate hierarchical analyses were performed using Poisson regression with a robust estimator.

**Results:**

The prevalence of allergic rhinitis symptoms was 21.7%. Children whose mothers had over 8 years of education, or who had air conditioning equipment in the house, or whose bedroom walls presented mold or moisture showed statistically significant and independent 5% higher prevalence of allergic rhinitis. Similarly, children of smoker mothers or those who lived with fur or feather animals indoors showed a 4% higher prevalence.

**Conclusion:**

Significant associations were observed between socio-demographic factors and environmental conditions in child's home and allergic rhinitis symptoms in children aged six years.

## RESUMO

**Objetivo:**

Estimar a prevalência de sintomas de rinite alérgica e fatores associados em crianças de seis anos de idade.

**Métodos:**

Estudo epidemiológico de delineamento transversal envolvendo 956 escolares de seis anos de idade do município de Palhoça, Santa Catarina, Brasil. Foram realizadas entrevistas domiciliares com as mães sobre as informações sociodemográficas e as condições ambientais da casa e foi aplicado o questionário ISAAC para sintomas de rinite alérgica. Análises bivariada e multivariada hierarquizada foram realizadas por meio da Regressão de Poisson com estimador robusto.

**Resultados:**

A prevalência de sintomas de rinite alérgica foi de 21,7%. Crianças cujas mães apresentavam escolaridade maior que oito anos ou com presença de aparelho de ar condicionado ou mofo/umidade nas paredes do quarto em que dormiam apresentaram prevalência 5% maior, estatisticamente significativa e independente, de rinite alérgica. Da mesma forma, crianças com mães tabagistas ou que conviviam com animais de pelo ou pena dentro de casa apresentaram prevalência 4% maior.

**Conclusão:**

Foram observadas associações significativas entre fatores sociodemográficos e condições ambientais da casa da criança e sintomas de rinite alérgica em crianças de seis anos de idade.

## INTRODUCTION

Rhinitis is a diffuse inflammation or dysfunction of nasal lining mucosa caused by the action of infectious, allergenic or hyperreactive agents. It is classified as allergic, non-allergic, infectious or mixed rhinitis^1^. Allergic rhinitis is defined as a chronic inflammatory disease mediated by immunoglobulin E (IgE), triggered by exposure to innocuous allergens/proteins, after their sensitization^2^. Typical symptoms include nasal congestion, anterior and posterior rhinorrhea, sneezing, nasal itching, and hyposmia. Ocular symptoms such as conjunctival hyperemia and ocular pruritus may also occur, and in this case, it is called allergic rhinoconjunctivitis^2^. Usually, the symptoms last two or more consecutive days for more than an hour on most days^1–3^.

Allergic rhinitis is classified based on exposure to allergens, duration and severity of symptoms^4^. Exposure to allergens can be seasonal, caused by different types of pollen, perennial, caused by allergens that exist throughout the year, or occupational, caused by allergens in the workplace. The duration may be intermittent or persistent. The severity is divided into mild when there is no impairment of daily activities or moderate-severe when associated with sleep disorders and or impairment of daily or school activities^5^.

Allergic rhinitis is a common disease, one of the ten main causes for seeking Primary Health Care (PHC)^2^. Given its high prevalence in urban populations, some authors call it the disease of modern civilization^6^.

Considering its incidence in childhood, it is marked as one of the most important public health problems in the world^7^. The first International Study of Asthma and Allergies in Childhood (ISAAC)^8^ showed that its prevalence is higher in developed countries, with regional variability, even within the same country^7,9^. The worldwide prevalence ranged from 2.2 to 14.6% among children aged 6 to 7 years^9^. The results of the ISAAC study in Brazil showed that the average prevalence was 25.7% in schoolchildren, the highest rate in the world^9,10^. The prevalence has increased over the years and is probably underestimated, as many individuals do not recognize the allergic rhinitis as a disease and do not seek medical attention. Health professionals can often neglect it as well^3,9^. Patients with allergic rhinitis may have a two-fold increase in medication costs and almost a two-fold increase in medical visits costs^11^. Also, self-medication has been positively associated with self-reported diagnosis in Brazil^12^.

Since it is a disease of multifactorial etiology, the allergic rhinitis in children is associated with several triggering factors. Risk factors include lifestyle, environment and genetics^4,11^. As it is unlikely that the expression of genetic mutations would contribute to the increased prevalence of allergic rhinitis, environmental factors may be the most important to explain this growth^11^. National studies enhance the participation of house mites as the main etiological agents, followed by cockroach allergens, pet epithelium (pet dander), and visible mold on the room walls. In the southern region of the country, pollen is important in sensitizing children^13,14^. It is fundamental to emphasize the role of mucosal irritants, with special emphasis on pollutants, such as tobacco smoke, passive smoking, and traffic exhaust fumes^3,6^.

In addition to the triggering factors, there are other pathologies that can be developed in association with allergic rhinitis, such as asthma^15^. Other associated symptoms include fatigue, attention deficits, learning difficulty, weak memory, and even depression^15^. Nasal obstruction resulting from allergic rhinitis has been shown to contribute to the development of sleep disorders, even with regular treatment^16^.

Considering these aspects, there is a need for studies that assess the prevalence of allergic rhinitis and associated factors, as they may allow for the designing of adequate public policies for the population. Knowing the local setting facilitates the improvement of primary care, including actions for health promotion, preventing, and treating the disease in children. To complement the data continuously produced by health information systems, it is also essential to have population-based surveys^17^. Nationwide population-based epidemiological data are extremely important for major public policies, but those at the local level have enormous potential for planning adequate health care for people in municipalities, where the Brazilian Unified Health System (SUS) is implemented. Thus, the aim of this study was to estimate the prevalence of allergic rhinitis symptoms and associated factors in six-year-old children in a city in southern Brazil.

## METHODS

This was a cross-sectional epidemiological study nested in a longitudinal study called *Coorte Brasil Sul* ^18^ that monitors schoolchildren, and their respective families in the city of Palhoça, Santa Catarina (SC).

The population consisted of children born in 2009, dwelling in Palhoça (SC) and enrolled in all 37 public and 19 private schools in 2015. Thus, they were six years old at the time of the study. For sample size calculation, the following parameters were used: population of 1,756 schoolchildren, unknown anticipated prevalence of allergic rhinitis (p=50%), 95% confidence level and 3% relative error, which generated a minimum sample of 664 children. Therefore, all children present in the *Coorte Brasil Sul* ^18^ database, with all the necessary information for the present study were included (n=956). Children who jointly met the following criteria were included: born in 2009, enrolled in public and private schools in 2015, and residing in Palhoça. Children from families whose language was not Portuguese were excluded from the study.

Data were collected by the team of researchers of *Coorte Brasil Sul* ^18^ and by community health agents trained and qualified for this purpose. The research team was formally trained through 30 hours of theoretical and practical activities. Interviews were carried out with the children's mothers at home to obtain socio-demographic and house environmental conditions information. ISAAC^19^ questionnaire was also applied to obtain information on allergic rhinitis.

The dependent variable was the mothers’ report on the allergic rhinitis symptoms according to the ISAAC question: “In the past 12 months, has your child had a problem with sneezing, or a runny, or blocked nose when he/she did not have a cold or the flu?” (yes or no). The independent socio-demographic variables were: child's gender (male or female), child's ethnicity (white or non-white), type of school the child was attending (public or private), mother's and father's education level (up to 8 years of completed study or more than 8 years), mother's and father's occupation (with or without income), and mother with a stable partner (yes or no). The independent variables associated with environmental conditions in the child's home were: smoker mother (yes or no), living with fur or feather animals indoors (yes or no), presence of mold or moisture on the child's bedroom walls (yes or no), presence of air conditioning unit, carpet or rug, curtains and plush toys in the child's bedroom (yes or no), and usage of feather pillows by the child (yes or no).

**Figure 1.**
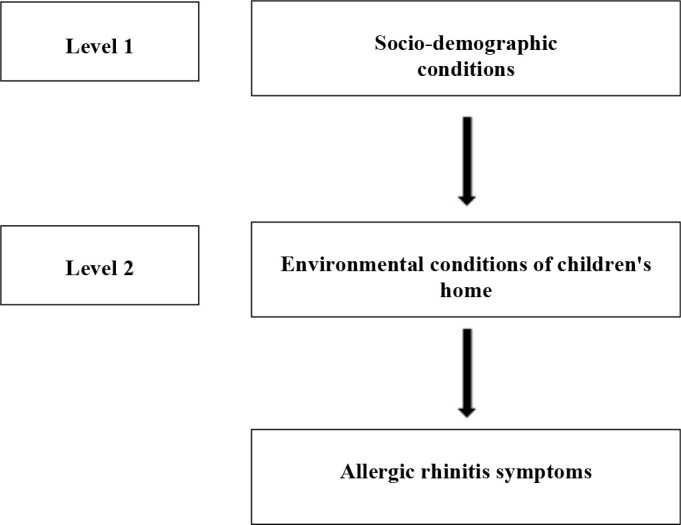
Hierarchical conceptual structure, in blocks, for reporting rhinitis symptoms at six years of age.

Data collected were exported to the Statistical Package for Social Sciences (SPSS) 18.0 software from Excel spreadsheets of the original database. They were then analyzed using hierarchical Poisson regression with a robust estimator with the stepwise forward strategy. Prevalence ratios (PR) and their respective 95% confidence intervals (95%CI) were estimated. The hierarchical model of analysis proposed for this study consists of two levels, and is shown in Figure 1. The socio-demographic variables constituted the first level and the variables related to the child's home environmental conditions, the second level. Initially, a bivariate analysis was carried out, with all the variables from each hierarchical level. A model was then created with variables of the first level that presented p<0.20. In this block, variables presenting p<0.05 were maintained. Afterwards, variables of the second level were added, which in the bivariate analysis presented p<0.20. At that time, variables of second level presenting p>0.05 were removed from the model. Socio-demographic variables that had shown statistical significance in the first stage of the multivariate model were maintained, regardless of the level of significance presented after the introduction of the variables associated with the child's home environmental conditions. Thus, a model with two levels was created. Variables from this second level presenting p<0.05 were kept in the model, without removing variables from the previous level. Therefore, a final model with two levels was obtained. The order of entering variables in each step followed the level of statistical significance observed in the bivariate analysis.

The present research project was submitted to and approved by the Ethics Committee for Research with Humans of the *Universidade do Sul de Santa Catarina* according to Opinion no. 38240114.0.0000.5369.

## RESULTS

Out of the 956 children included in the study, 21.7% (95%CI 19.1–24.3) presented symptoms of allergic rhinitis. The results obtained in the bivariate analysis among socio-demographic variables and environmental conditions in the child's home and the symptoms are shown in Tables 1 and 2.

**Table 1:** Results of the bivariate analysis between socio-demographic variables and rhinitis symptoms at six years of age. Palhoça (SC), Brazil.

Variables	Allergic rhinitis symptoms				
	n	%	PRc	95%CI	p
Child's gender (n=956)					
Male	491	51.4	1.00		0.042
Female	465	48.6	0.97	0.94–0.99	
Child's ethnicity (skin color) (n=952)					
Caucasian	743	78.0	1.00		0.640
Non-caucasian	209	22.0	1.01	0.97–1.05	
Child's school type (n=953)					
Public	790	82.9	1.00		0.057
Private	163	17.1	1.04	1.01–1.09	
Mother's education (years of completed schooling) (n=919)					
Up to 8	499	54.3	1.00		<0.001
More than 8	420	45.7	1.06	1.02–1.09	
Father's education (years of completed schooling) (n=792)					
Up to 8	430	54.3	1.00		<0.001
More than 8	362	45.7	1.06	1.03–1.10	
Mother's occupation (n=938)					
No income	361	38.5	1.00		0.064
With income	577	60.2	1.03	0.99–1.06	
Father's occupation (n=876)					
No income	51	5.8	1.00		0.742
With income	825	94.2	1.01	0.94–1.08	
Mother with stable partner (n=948)					
Yes	783	82.6	1.01	0.97–1.05	0.642
No	165	17.4	1.00		

PRc: crude prevalence ratio; 95%CI: 95% confidence interval; p: p-value obtained by Poisson regression with robust estimator.

**Table 2:** Results of the bivariate analysis between environmental conditions in the child's home and rhinitis symptoms at six years of age. Palhoça (SC), Brazil.

Variables	Allergic rhinitis symptoms				
	n	%	PRc	95%CI	p
Smoker mother (n=948)					
Yes	171	18.0	1.04	1.01–1.08	0.016
No	777	82.0	1.00		
Living with fur or feather animals indoors (n=957)					
Yes	479	50.1	1.04	1.01–1.07	0.004
No	478	49.9	1.00		
Presence of mold or moisture on the walls of the child’s room (n=956)					
Yes	159	16.6	1.04	1.01–1.09	0.043
No	797	83.4	1.00		
Air conditioning unit in the child’s room (n=957)					
Yes	240	25.1	1.06	1.03–1.11	0.001
No	717	74.9	1.00		
Tapestry or carpet in the child's room (n=958)					
Yes	325	33.9	1.01	0.99–1.04	0.508
No	633	66.1	1.00		
Curtain in the child's room (n=958)					
Yes	808	84.3	1.02	0.99–1.07	0.259
No	150	15.7	1.00		
Sleeping with feather pillow (n=949)					
Yes	38	4.0	1.01	0.93–1.09	0.776
No	911	96.0	1.00		
Child's bedroom stuffed animal (n=958)					
Yes	539	56.3	1.02	0.99–1.05	0.183
No	419	43.7	1.00		

PRc: crude prevalence ratio; 95%CI: 95% confidence interval; p: p-value obtained by Poisson regression with robust estimator.

The final model obtained in the hierarchical multivariate analysis is shown in Table 3. Variables associated with 5% significant and independent higher prevalence of symptoms of rhinitis were: mother's educational level greater than 8 years (PR=1.05; 95%CI 1.02–1.08) (p-value [p]=0.001); presence of an air conditioning unit in the child's room (PR=1.05; 95%CI 1.01–1.09) (p=0.014); and child living with fur or feather animals indoors (PR=1.05; 95%CI 1.01–1.08) (p=0.003). Variables associated with 4% significant and independent higher prevalence of symptoms of rhinitis were maternal smoker (PR=1.04; 95%CI 1.01–1.07) (p=0.044), and presence of mold or moisture on the walls of the child's room (PR= 1.04; 95%CI 1.01–1.09) (p=0.050).

**Table 3:** Final hierarchical model for symptoms of allergic rhinitis at six years of age. Palhoça (SC), Brazil.

Variables	Allergic rhinitis symptoms		
	PRa	95%CI	p
1st level – socio-demographic conditions			
Mother's education (years of schooling completed)			
Up to 8	1.00		0.001
More than 8	1.05	1.02–1.08	
2nd level – environmental conditions of children's home			
Smoker mother			
Yes	1.04	1.01–1.07	0.044
No	1.00		
Living with fur or feather animals indoors			
Yes	1.05	1.01–1.08	0.003
No	1.00		
Air conditioning unit in the child's room			
Yes	1.05	1.01–1.09	0.014
No	1.00		
Presence of mold or moisture on the walls of the child's room			
Yes	1.04	1.01–1.09	0.050
No	1.00		

PRa: adjusted prevalence ratio; 95%CI: 95% confidence interval; p: p-value obtained by Poisson regression with robust estimator.

## DISCUSSION

In addition to the high prevalence (21.7%), this study showed significant associations between sociodemographic factors and environmental conditions in the child's home and symptoms of allergic rhinitis. These population-based results are a contribution to the national literature about the constant need to update epidemiological data in Brazil, a country of continental dimensions, with marked climatic, sociocultural, and economic differences. In India, another country with large dimensions and inequalities, the prevalence of allergic rhinitis among the 6–7 years age group was 11.3%^20^.

Back to Brazil, the ISAAC study Phase 3^21^ found a national mean prevalence, in this same age group in 2002–2003 of 25.7%, similar to that of the present study. Furthermore, the prevalence in the southern region of Brazil ranged from 20.6 to 39.2%. However, there was a decrease in the prevalence of allergic rhinitis diagnosis between 2011 (50.0%) and 2018 (30.0%) in the age group 0–9 years in a municipality of Rio Grande do Sul^22,23^.

Allergic rhinitis is a multifactorial disease^24^, which is why the hierarchical variable analysis model was used in this study. It is an IgE-mediated type 1 hypersensitivity illnesses triggered by a spectrum of environmental allergens from outdoor origin like pollen, and from indoor origin, like arthropod or mammalian derived allergens such as dust mites, cockroaches, cat allergens or molds^25^.

After evaluating the variables included in this study, it was found that higher maternal education, smoker mother, living with fur or feather animals indoors, air conditioning unit in the child's bedroom and the presence of mold or moisture on the walls of the child's bedroom were independently associated with a higher prevalence of allergic rhinitis symptoms.

The mother's higher education level was associated with a 5% higher prevalence. Since this is an indicator of socioeconomic development, something that could justify it is the Hygiene Hypothesis^26^, which suggests that for the development of the immune system, prior exposure to pathogenic agents that protect against the development of allergic diseases is necessary. However, in developed societies there are factors that limit children's contact with different pathogens and prevent the manifestation of acute infectious diseases in early childhood, inhibiting the action of Th1 lymphocytes and favoring the activation of Th2 lymphocytes, responsible for chronic allergic manifestations^26^.

Nevertheless, the fact that the mother's higher education is associated with the presence of rhinitis symptoms could be an indicative of greater access to products, such as air conditioning unit, curtains and rugs, in addition to the presence of pets at home. In this context, it can be assumed that social status might be a proxy measure for behavioral patterns^27^. Loo et al.^28^ also found that higher maternal education was a risk factor for allergic rhinitis. These results agree with a systematic review of 183 studies showing a higher prevalence of rhinitis in higher socioeconomic status groups^29^.

Regarding smoker mothers, there was a 4% higher prevalence of allergic rhinitis, corroborating the studies that confirm passive smoking as a triggering factor for chronic and respiratory diseases, with children being the most affected, as they spend more time exposed to fumes, especially when the mother or caregiver are the ones who smoke^20,30^. A study confirmed that maternal smoking was the strongest of all the associated features for allergic rhinitis, rhinoconjunctivitis, and eczema, especially in the 6-7 years age group^20^. Passive smoking was also found to be associated with allergic rhinitis in a case-control study in a hospital in Jiangxi^31^ as well as a risk factor for allergic rhinitis in children confirmed by a recent meta-analysis^32^, both in China.

Children living in contact with fur or feather animals indoors were associated with a 5% higher prevalence of allergic rhinitis symptoms compared to those not living with this kind of animals. This can be a triggering factor for allergic processes^27^. However, a study^27^ showed that the association between animal contact and allergic sensitization, atopic diseases or their symptoms are not homogeneous across social strata. The authors showed that cat contact was significantly associated with an increased odds of sensitization to cat only in children whose parents have a high level of education.

The presence of an air conditioning unit in the child's bedroom was also associated with a higher prevalence of allergic rhinitis. A study carried out with adults also found a higher prevalence of respiratory diseases in those children exposed to air conditioning units. The literature pointed out that the lack of proper maintenance of such equipment generates an accumulation of allergens such as fungi, animal hair and dust mites in the environment^14,25^, which could also be associated with an increased prevalence of the condition in the child population.

Another associated variable is the presence of mold or moisture on the child's bedroom walls. Recent data have shown the association between exposure to fungi in early childhood and the development of atopic conditions, including asthma, in late childhood^33,34^. Studies^35,36^ observed that humidity in classrooms was strongly associated with an increase in wheezing crises and a decrease in spirometry among students exposed to humid settings. There is sufficient evidence for the associations between moisture/mold damages and different health effects such as allergic respiratory diseases, asthma, allergic rhinitis, exogenous allergic alveolitis and respiratory tract infections/bronchitis. However, in comparison to other environmental allergens, the sensitizing potential of molds is estimated to be low^37^. These data indicate that it is important to investigate in cases of patients with anamnesis of respiratory allergies the possibility of exposure to fungi not only at home but also in day care centers, schools, and workplaces.

The present study has some limitations requiring caution in the analysis of the results presented. As ISAAC is a questionnaire applied to the mother, it is possible to admit eventually, the occurrence of memory bias. Also, as allergic rhinitis is an outcome self-reported by the mother, it can eventually be inferred that the relationship between higher education and better socioeconomic conditions is influencing a greater detection and reporting of rhinitis symptoms. Likewise, the use of the ISAAC does not allow for the diagnosis of rhinitis, but for the reporting of symptoms. However, most epidemiological studies have been performed in school-age children using the ISAAC questionnaire^28^.

This study reveals that allergic rhinitis is highly prevalent among children in the studied city, resulted from a complex interplay of socio-demographic and environmental factors. Due to the cross-sectional nature of the current study, it was only possible to evaluate associations and not etiologic relationships. It is necessary to plan further prospective analytical studies in order to establish the role of risk factors as predictors for allergic rhinitis in children in different regions and social strata in Brazil, providing basis for planning public health interventions. Public health measures may be outlined to combat these associated environmental factors and provide access to medical care to manage atopic disorders. In times of the COVID-19 pandemic, it is relevant to mention the need for managers and health professionals to know the possible relationship of symptoms between the two diseases^38^.

In conclusion, significant associations were observed between socio-demographic factors and environmental conditions in the child's home and symptoms of allergic rhinitis in children aged six in the city studied.
